# Saikosaponin d Alleviates Liver Fibrosis by Negatively Regulating the ROS/NLRP3 Inflammasome Through Activating the ERβ Pathway

**DOI:** 10.3389/fphar.2022.894981

**Published:** 2022-05-25

**Authors:** Kehui Zhang, Liubing Lin, Yingying Zhu, Na Zhang, Meng’en Zhou, Yong Li

**Affiliations:** Department of Gastroenterology, Shanghai Municipal Hospital of Traditional Chinese Medicine, Shanghai University of Traditional Chinese Medicine, Shanghai, China

**Keywords:** saikosaponin d, estrogen receptors, mitochondrial reactive oxygen species, nodlike receptor protein 3 inflammasome, liver fibrosis

## Abstract

**Background and aims:** Saikosaponin d (SSd) has a steroidal structure and significant anti-inflammatory effects. The purpose of this study was to explore the mechanism underlying SSd’s inhibitory effects on liver fibrosis.

**Methods:** Wild-type and estrogen receptor knockout (ERKO) mice were treated with CCl_4_ to establish liver fibrosis mouse models. The effects of SSd on hepatic fibrogenesis were studied in these mouse models. Hepatic stellate cells (HSCs) were activated by H_2_O_2_ to investigate the potential molecular mechanisms. The establishment of the models and the degrees of inflammation and liver tissue fibrosis were evaluated by detecting changes in serum liver enzymes and liver histopathology. The expression of α-SMA and TGF-β1 was determined by immunohistochemistry. The expression and significance of NLRP3 inflammasome proteins were explored by RT-PCR and Western blotting analyses. The mitochondrial ROS-related indexes were evaluated by MitoSOX Red.

**Results:** In wild-type and ERKO mice treated with CCl_4_, the fluorescence expression of mitochondrial ROS was up-regulated, while the mitochondrial membrane potential and ATP content were decreased, suggesting that the mitochondria were damaged. In addition, the expression of NLRP3 inflammatory bodies and fibrosis markers (α-SMA, TGF-β, TIMP-1, MMP-2, and Vimentin) in liver tissue increased. Furthermore, the above indexes showed the same expression trend in activated HSCs. In addition, the peripheral serum ALT and AST levels increased in CCl_4_-induced liver injury model mice. And HE staining showed a large number of inflammatory cell infiltration in the liver of model mice. Picric acid-Sirius staining and Masson staining showed that there was significant collagen fibrous tissue deposition in mice liver sections. IHC and WB detection confirmed that the expression of α-SMA and TGF-β1 increased. Liver fibrosis scores were also elevated. Then, after SSd intervention, the expression of ROS in wild-type mice and αERKO mice decreased, mitochondrial membrane potential recovered, ATP level increased, NLRP3 inflammasome and fibrosis indexes decreased, liver enzyme levels decreased, and liver pathology showed liver inflammation. The damage and collagen deposition were significantly relieved, the expression of α-SMA and TGF-β1 was decreased, and the fibrosis score was also decreased. More importantly, the effect of SSd in alleviating liver injury and liver fibrosis had no effect on βERKO mice.

**Conclusion:** SSd alleviated liver fibrosis by negatively regulating the ROS/NLRP3 inflammasome through activating the ERβ pathway. By establishing liver fibrosis models using wild-type and ERKO mice, we demonstrated that SSd could alleviate liver fibrosis by inhibiting the ROS/NLRP3 inflammasome axis through activating the ERβ pathway.

## Introduction

Liver fibrosis is a pathological process through which various chronic liver diseases develop into liver cirrhosis (Pinzani, 2015). Epidemiological studies have shown that various pathogenic factors can trigger liver inflammation, leading to liver cell damage, abnormal proliferation of connective tissue, reduced liver elasticity, and ultimately deteriorated liver tissue structure and functions (Fattovich et al., 2004; Pinzani, 2015; Parola and Pinzani, 2019). Liver fibrosis is reversible. However, once liver cirrhosis develops, liver fibrosis becomes irreversible. Therefore, blocking or reversing liver fibrosis is essential for the treatment of chronic liver diseases (Friedman, 2007; Böttcher and Pinzani, 2017; Parola and Pinzani, 2019). At present, the treatments for liver fibrosis mainly include anti-inflammation, anti-fibrosis, liver protection and other therapies. Although considerable effort has been spent on further understanding the mechanisms of current treatments and exploring effective novel treatments, satisfactory results have not been obtained (Chen et al., 2015; Böttcher and Pinzani, 2017). None of the new methods developed so far is perfect and all of them are far from ready for clinical practice. Therefore, there is still an urgent need to develop novel methods to block or reverse liver fibrosis.

The activation of hepatic stellate cells (HSCs) is a key step during liver fibrosis, which can lead to the production of a large amount of ROS from mitochondria. Overproduction of ROS can in turn promote the activation of HSCs and the NLPR3 inflammasome (Mederacke et al., 2013). According to previous studies, the NLRP3 inflammasome is expressed in many types of liver cells, including HSCs and damaged hepatocytes (Ning et al., 2017; Kim et al., 2018; Mohamed et al., 2018). Anomalous NLRP3 inflammasome activation is associated with the development of many diseases (Zahid et al., 2019). Oxidative stress injury of Mitochondria and activation of NLRP3 inflammatory body play an important role in the development of liver fibrosis. Key components of a functional NLRP3 inflammasome are NLRP3, the adaptor protein ASC and caspase-1. When oxidative stress occurs, NLRP3 recruits ASC and Pro-caspase-1, resulting in activation of Caspase-1 and prohibiting cytokines IL-1β and IL-18 (Martinon et al., 2002; Zahid et al., 2019). It has been widely documented that ROS play an important role in upstream signaling pathways that activate NLRP3 inflammasome (Mederacke et al., 2013; Abais et al., 2015). Recent literature found that NLRP3 inflammasome activation induced by mitochondrial oxidative stress plays an important role in the development of liver fibrosis (Zhao et al., 2019). Physiologically, quiescent HSCs are required for extracellular matrix (ECM) remodeling. Once activated, quiescent HSCs turn into myofibroblasts, accompanied by the overexpression and deposition of ECM. MMP and TIMP proteins play an important role in the process of liver fibrosis and activation of HSCs by regulating the homeostasis and remodeling of ECM. The levels of MMP-2 and TIMP-1 increase during those processes. Studies have confirmed that a reduction in E-Cadherin expression can promote the development of liver fibrosis, renal interstitial fibrosis, peritoneal fibrosis, and pulmonary fibrosis in experiments (Lu et al., 2018). Vimentin, which is mainly expressed in mesenchymal cells, is an intermediate filament protein and an important biomarker of epithelial–mesenchymal transition (EMT) (Cheng et al., 2016). EMT sustains the generation of pathologically “activated” ATII cells that, by secreting pro-fibrotic factors, further amplifies the fibrotic response (Ruaro et al., 2021). When HSCs were activated by lipopolysaccharide (LPS) and H_2_O_2_, the expression of NLRP3 inflammatory bodies in HSCs increased (Lin et al., 2018; Zahid et al., 2019).

Bupleurum is a traditional Chinese medicine that has been used for the treatment of liver disorders and other common diseases in China for centuries. As an active component of Bupleurum, saikosaponin d (SSd) plays a significant role in anti-inflammation, anti-liver fibrosis, anti-lipid peroxidation, inhibiting the activation of HSCs, and reducing matrix production (Dang et al., 2007; Fan et al., 2007). SSd is a potential treatment for liver fibrosis. Our previous research proved that SSd has estrogenic effects *in vivo* and *in vitro* and is an estrogen receptor (ER) modulator (Que et al., 2018). In addition, no obvious hepatotoxicity was observed for SSd at an effective drug dose (Wang et al., 2010). Research on the anti-fibrosis effects of estrogen confirmed that ERs are expressed in hepatocytes, sinusoidal endothelial cells, Kupffer cells and stellate cells, and that estrogen can improve the microcirculation of damaged liver, inhibit the proliferation of activated HSCs and the synthesis of collagen, promote the activity of antioxidant enzyme GPx in hepatocytes, prevent lipid peroxidation, regulate the expression of cytokines such as transforming growth factor, and inhibit the occurrence and development of liver fibrosis (Zhang et al., 2016; Lee et al., 2019). Studies have shown that the inhibition of NLRP3 inflammasome depends on the blockade of ERα in colitis (Gao et al., 2020). Zhang et al. demonstrated that estrogen exerted its anti-fibrosis effects via ERβ rather than ERα or GPER (Zhang et al., 2018). However, it is unclear whether SSd or estrogen alleviates liver fibrosis by inhibiting the expression of NLRP3 inflammasome proteins via activating the ERβ pathway.

In this study, we established models of CCl_4_-induced liver injure and fibrosis in wild-type and ERKO mice to investigate the molecular mechanism underlying SSd’s anti-fibrosis effects. Specifically, we focused on the expression of protein components of the NLRP3 inflammasome and the role of ERs.

## Materials and Methods

### Animal Experiments

Male specific-pathogen-free (SPF) C57BL/6 mice (6–8 weeks, 20 ± 2 g) were purchased from Shanghai SLAC Laboratory Animal Co., Ltd (Shanghai, China). After purchase, the mice were raised in the animal room without specific pathogens (SPF) in Shanghai Municipal Hospital of Traditional Chinese Medicine. With 5 mice in each cage, they were allowed free access to foods and drinks. Throughout 1 week when the mice were fed adaptively, the temperature was maintained at 20–25°C and the humidity was restricted to the range of 40–70%. All the animal experiments were performed in compliance with local and national guidelines. These experiments approved by the animal experimental ethics and welfare committee of Shanghai Municipal Hospital of Traditional Chinese Medicine (No. 2015032).

For establishment of liver fibrosis mouse models, we injected male wild-type and ER-α and -β knockout mice (αERKO and βERKO mice, respectively, TOS160106CC1, Cyagen Biosciences) subcutaneously twice weekly for 6 weeks with 5 ml/kg body weight carbon tetrachloride (CCl_4_) diluted 1:4 in vegetable oil. Mice injected with only vegetable oil served as a vehicle control group. To investigate the effect of SSd and the relationship between mitochondrial ROS level and the expression of NLRP3 inflammasome proteins, C57BL/6 male mice were injected subcutaneously with SSd (China National Institute for the Control of Pharmaceutical and Biological Products, China) at a dosage of 2 mg/kg body weight twice a week for 6 weeks. Thus, the following groups (10 mice per group) were established in this study (Pinzani, 2015): vehicle control group (Fattovich et al., 2004); CCl_4_-induced hepatic fibrosis model group (Parola and Pinzani, 2019); CCl_4_+SSd group: hepatic fibrosis mice treated with SSd.

### Serum Biochemical Analyses

Pre-treatment of samples for determination of serum ALT and AST levels: About 500 µl blood was collected from eyeballs of mice and placed in a 1.5 ml apical centrifuge tube at room temperature for 1–2 h, then centrifuged at 4°C at 3,000 rpm for 10 min. The upper serum was collected and placed in a new centrifuge tube for analysis, or stored in a −80°C freezer. ALT and AST analysis kits (Nanjing Jiancheng Biology Research Institute Co., Ltd.) were used to determine their serum levels.

### Histological Staining and Determination of the Non-Alcoholic Fatty Liver Disease Fibrosis Score

Liver tissue sections were prepared, fixed in 4% paraformaldehyde at room temperature overnight, and subjected to hematoxylin-eosin (H&E) staining, Masson’s trichrome staining and picric acid-Sirius red staining. The stage of liver fibrosis was assessed according to the Ishak score.

### Immunohistochemical (IHC) Analysis of the Expression of α-SMA and TGF-β1

Paraffin-embedded sections were baked in a thermostat for 2 h, dewaxed and rehydrated with xylene and ethanol, incubated in 3% H_2_O_2_ solution at room temperature for 10 min to block endogenous peroxidase activity, antigen-retrieved with citric acid buffer, incubated for 30 min with 2% goat serum, incubated with primary antibodies against α-SMA and TGF-β1 overnight at 4°C, washed with PBS, incubated with polymer auxiliaries 20 min at 37°C, and incubated with a biotinylated secondary antibody for 2 h at room temperature. The sections were visualized with DBA and the nuclei were stained with hematoxylin. Sectioning, dehydration and mounting were performed according to routine protocols and the sections were observed under a microscope (AE41, McAudi Industrial Group Co., Ltd.).

### Cell Culturing and Treatment

HSCs-LX2 (Shanghai Fanling Biotechnology Co., Ltd., Shanghai, China) were cultured in DMEM at 37°C with 5% CO_2_. After seeding in 6 well plates, HSCs were treated with H_2_O_2_ at a concentration of 200 μmol/L for 4 h. To investigate the role of ERs in SSd’s anti-fibrotic effects, HSCs-LX2 were treated with MPP (1 μM) (Tocris Bioscience Co., Ltd. UK) or THC (1 μM) (Tocris Bioscience Co., Ltd. UK) for 30 min, subsequently with SSd (5 μM) for 24 h, and finally incubated with H_2_O_2_ (200 μM) for 4 h in complete DMEM.

### Protein Extraction and Western Blotting Analysis

After being extracted from liver tissues and cells, protein samples were separated with SDS-PAGE, and electroblotted onto polyvinylidene difluoride membranes. After blocking with 5% skimmed milk, the membranes were incubated with primary antibodies, then with an appropriate horseradish peroxidase-labeled secondary antibody (1:2000; CST signaling, United States). Specific bands were detected by an enhanced chemiluminescence assay. Primary antibodies and their working concentrations are listed in Supplementary Table S1.

### RNA Isolation and Real-Time Polymerase Chain Reaction (RT-PCR)

The TRIzol (Invitrogen,United States) reagent was applied for total RNA extraction. Reverse transcription and quantitative RT-PCR were performed with the PrimeScript® RT reagent kit (TaKaRa, Japan) and SYBR Premix Ex Taq (TaKaRa, Japan). The primers were synthesized by Shanghai Shenggong Biology Co., Ltd. Expression levels of genes relative to β-actin mRNA levels in each sample were calculated according to the 2^−ΔΔCt^ method. The primer sequences are shown in Supplementary Table S2, S3.

### Determination of Mitochondrial ROS Level Using MitoSOX Red

To determine the level of mitochondrial ROS, a working solution of the probe (5 μM, Beyotime Institute of Biotechnology) was added into cells and incubated in the dark. The cells were washed with preheated PBS, re-stained with a working solution of Hoechst33342 (10 μg/ml, Beyotime Institute of Biotechnology), washed with PBS again, and observed under a fluorescence microscope (DMI3000B, Leica Corp.).

The mitochondria isolation solution was added to liver tissues for grinding. The liquid phase was centrifuged, and the precipitate, which contained mitochondria from liver tissues, was retained. The precipitate was resuspended with mitochondria storage solution, mixed with MitoSOX Red (5 μM, Beyotime Institute of Biotechnology), and incubated at 37°C for 30 min. The fluorescence intensity was determined by a fluorescence microplate reader (DMI3000B, Leica Corp.).

### Evaluation of HSCs-LX2 Viability and Lipid Peroxidation

HSCs-LX2 were seeded into 96-well plates at a density of 1 × 10^4^ cells per well and cultured in an incubator at 37°C with 5% CO_2_. H_2_O_2_ (at a concentration of 200 μM) was added into the cells for a 4-h incubation. The viability of HSCs-LX2 was determined by CCK-8 assays (Shanghai Yisheng Bio, China). MDA is an important product of lipid peroxidation, the level of which reflects the degree of lipid peroxidation. Therefore, we evaluated the degree of lipid peroxidation using an MDA assay kit (Beyotime Institute of Biotechnology) according to the manufacturer’s instructions.

### Measurement of Mitochondrial Membrane Potential

The JC-1 probe (Beyotime Institute of Biotechnology) was used for mitochondrial membrane potential measurement. Briefly, cells were treated with JC-1 probe monomers according to the manufacturer’s instructions. Then, nuclei were visualized by DAPI staining (Beyotime Institute of Biotechnology) in the dark. The ratio of red and green fluorescence signal intensities of JC-1 probe monomers was calculated.

### Intracellular Adenosine Triphosphate (ATP) Level Assay

Intracellular ATP levels were measured with an assay kit (Beyotime Institute of Biotechnology) in accordance with the manufacturer’s instructions.

### Data Presentation and Statistical Analyses

All data are expressed as means ± SEM. Student’s t-test was used to compare the differences between the means of two groups. One-way analysis of variance (ANOVA) with post hoc Fisher’s least significant difference tests was performed for comparisons among multiple groups using SPSS 22.0 (SPSS Inc., Chicago, United States). A *p* value of less than 0.05 was considered statistically significant.

## Results

### SSd Alleviated CCL_4_-Induced Liver Injury and Liver Fibrosis in Mice

**FIGURE 1:**
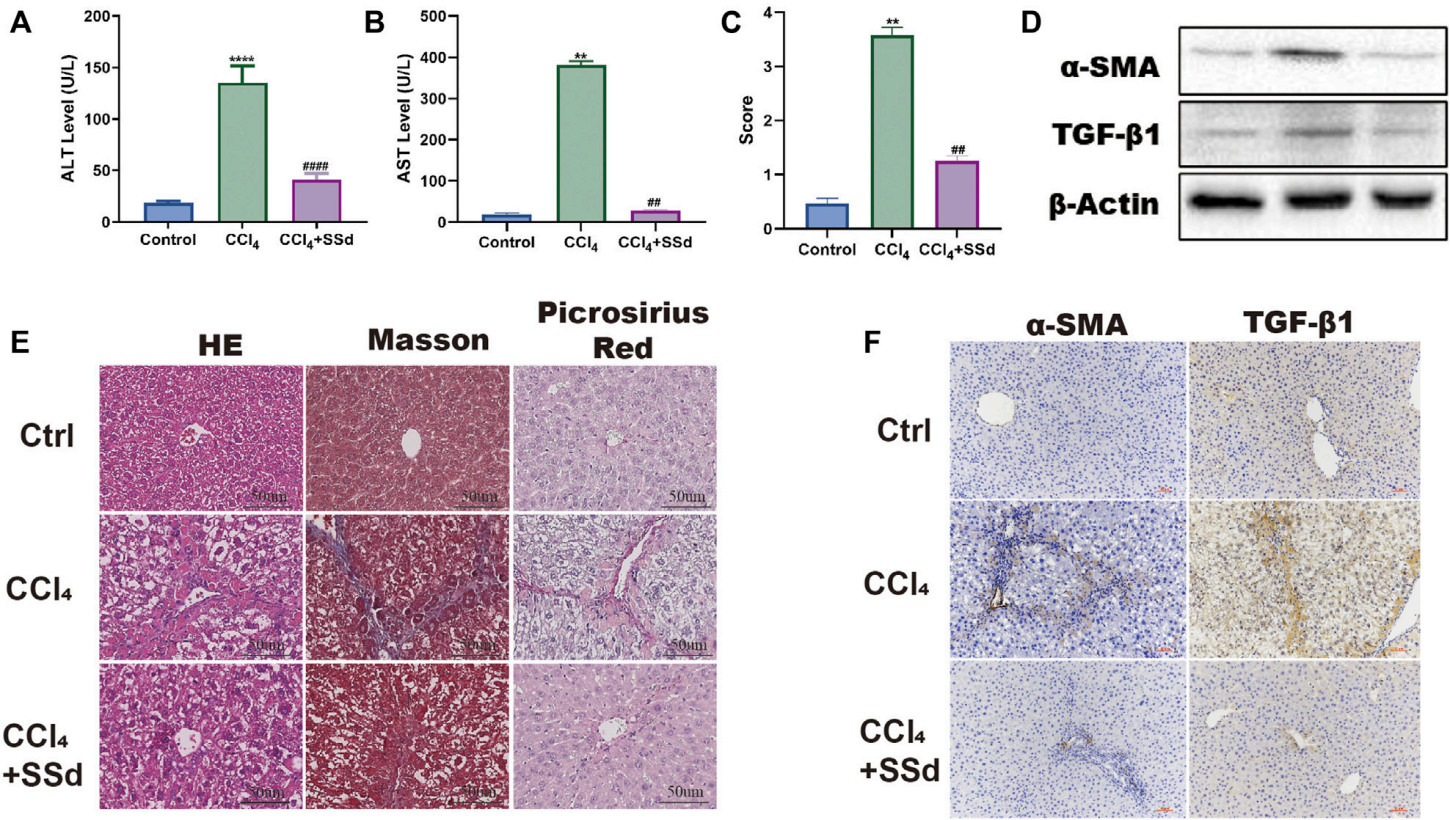
SSd alleviated CCL_4_-induced liver injury and liver fibrosis. **(A,B)** the changes of serological ALT and AST concentration, *n* = 6. **(C)** Liver Fibrosis Score, *n* = 6. **(D–F)** liver histological determination (H&E) and IHC of α-SMA and TNF-β (×200; scale bar: 50 μm). Data are expressed in mean ± SEM; **p < 0.01, ****p < 0.0001, versus control group; ##*p* < 0.01, ####*p* < 0.0001, AND versus CCL_4_ treated group.

To investigate the effect of SSd on liver injury, we established a mouse model of CCL_4_-induced liver injury and liver fibrosis. Unlike to the control group, the AST and ALT levels of the model group increased significantly (Figures 1A,B). The liver fibrosis score of the model group was also meaningfully higher than that of the control group (Figure 1C). Liver injury and fibrosis was occurred as shown by H&E staining (inflammatory cell infiltration and lobular disorder), Masson’s trichrome staining (blue color was indicative of collagen deposition), Picrosirius red staining (red color was indicative of collagen deposition) and the IHC of α-SMA and TNF-β1 (Figures 1D–F). On the contrary, the intervention of SSd significantly promoted the recovery of liver function and the normal liver structure. First of all, we also observed that the activities of liver enzymes AST and ALT were lower in the SSd group than in the control group, indicating an improved liver function (Figures 1A,B). Furthermore, the liver fibrosis score also decreased in the treatment group compared with the model group (Figure 1C). H&E staining displayed significantly reduced proliferation of fibrous tissues, significantly decreased inflammatory cell infiltration, and not obvious fat infiltration in the liver of model mice treated with SSd (Figure 1E). Masson staining revealed that the deposition of collagen fibers was reduced in the SSd intervention group (Figure 1E). Picric acid-Sirius red staining exhibited only a few collagen fibers in the vascular wall of the portal area after SSd intervention (Figure 1E). Correspondingly, the expression levels of α-SMA and TGF-β1 in the portal area and liver tissues of the SSd intervention group were significantly decreased compared with the model group (Figures 1D,E), suggesting that SSd treatment reduced hepatic inflammation and damages in the model mice.

### SSd Inhibited Mitochondrial Dysfunctions and NLRP3 Inflammasome Activation in Mice

**FIGURE 2:**
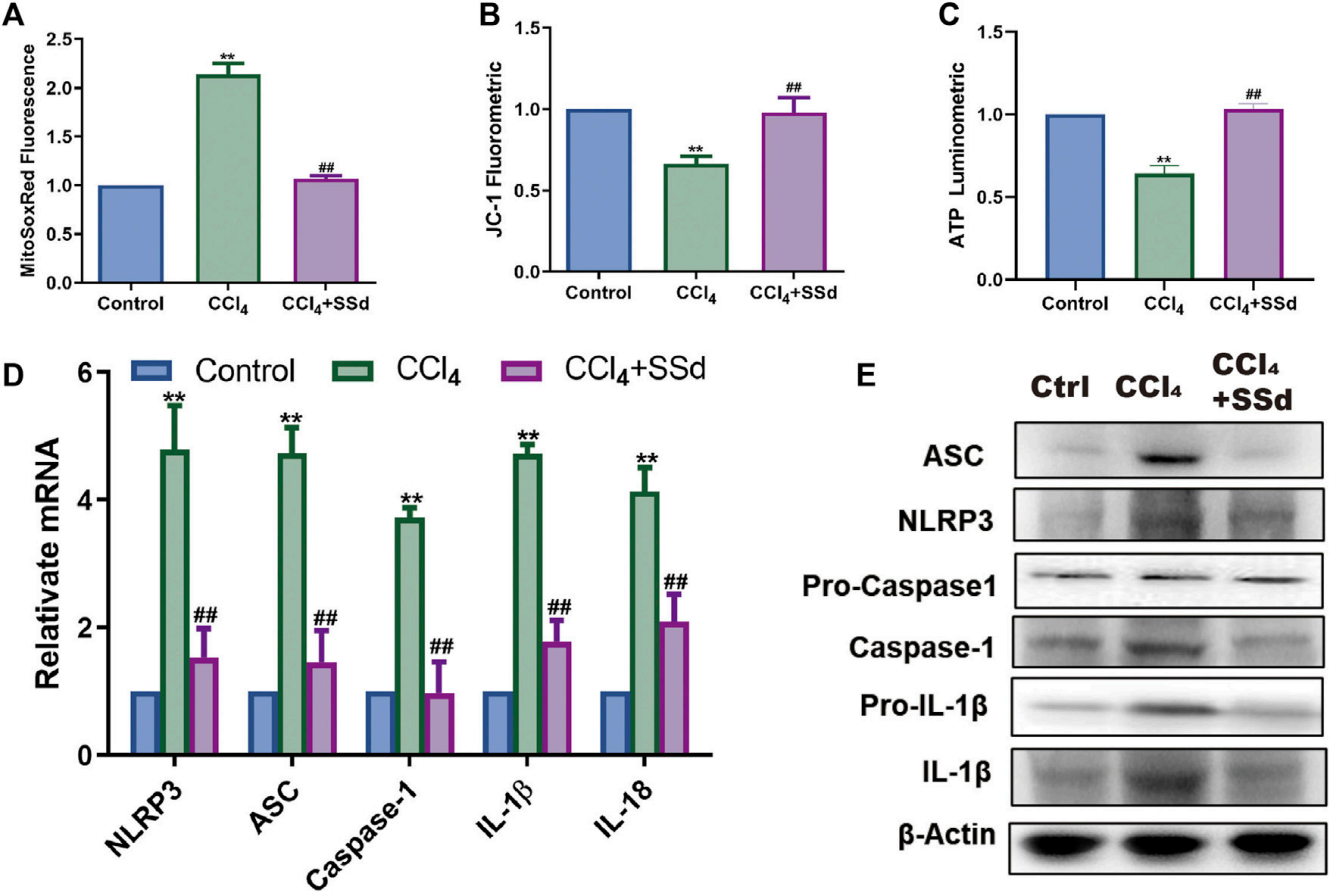
SSd inhibited mitochondrial dysfunctions and NLRP3 inflammasome activation. **(A)** MitoSOX Red Mitochondrial Superoxide Indicator detection, *n* = 6. **(B)** mitochondrial JC-1 fluorescence expression, *n* = 6. **(C)** ATP content, *n* = 6. **(D,E)** relative mRNA and proteins expression of inflammasome, *n* = 3. Data are expressed in mean ± SEM; **p < 0.01, CCL_4_ group versus control group; ##*p* < 0.01, CCl_4_+SSd versus CCL_4_ treated group.

Compared with untreated mice, the intensity of MitoSOX Red fluorescence in the SSd intervention group was decreased (Figure 2A). Determination of mitochondrial membrane potential showed that the ratio of JC-1 monomers with red fluorescence was higher in the SSd group than in the model group, and a similar trend was observed for intracellular ATP content (Figures 2B,C). As illustrated in Figures 2D,E, the expression levels of NLRP3 inflammasome, pro-IL-1β, IL-1β, and IL-18 in model mice were decreased significantly after treatment with SSd. Altogether, these data suggested that the effect of SSd on NLRP3 inflammasome activation in liver fibrosis was likely mediated through the regulation of mitochondrial ROS level. Taken together, these data suggested that SSd had inhibitory effects on the activation of the NLRP3 inflammatory proteins and mitochondrial oxidative stress damage in a model of liver fibrosis.

### SSd Alleviated H_2_O_2_-Induced Mitochondrial Dysfunctions and NLRP3 Inflammasome Activation in HSCs

**FIGURE 3:**
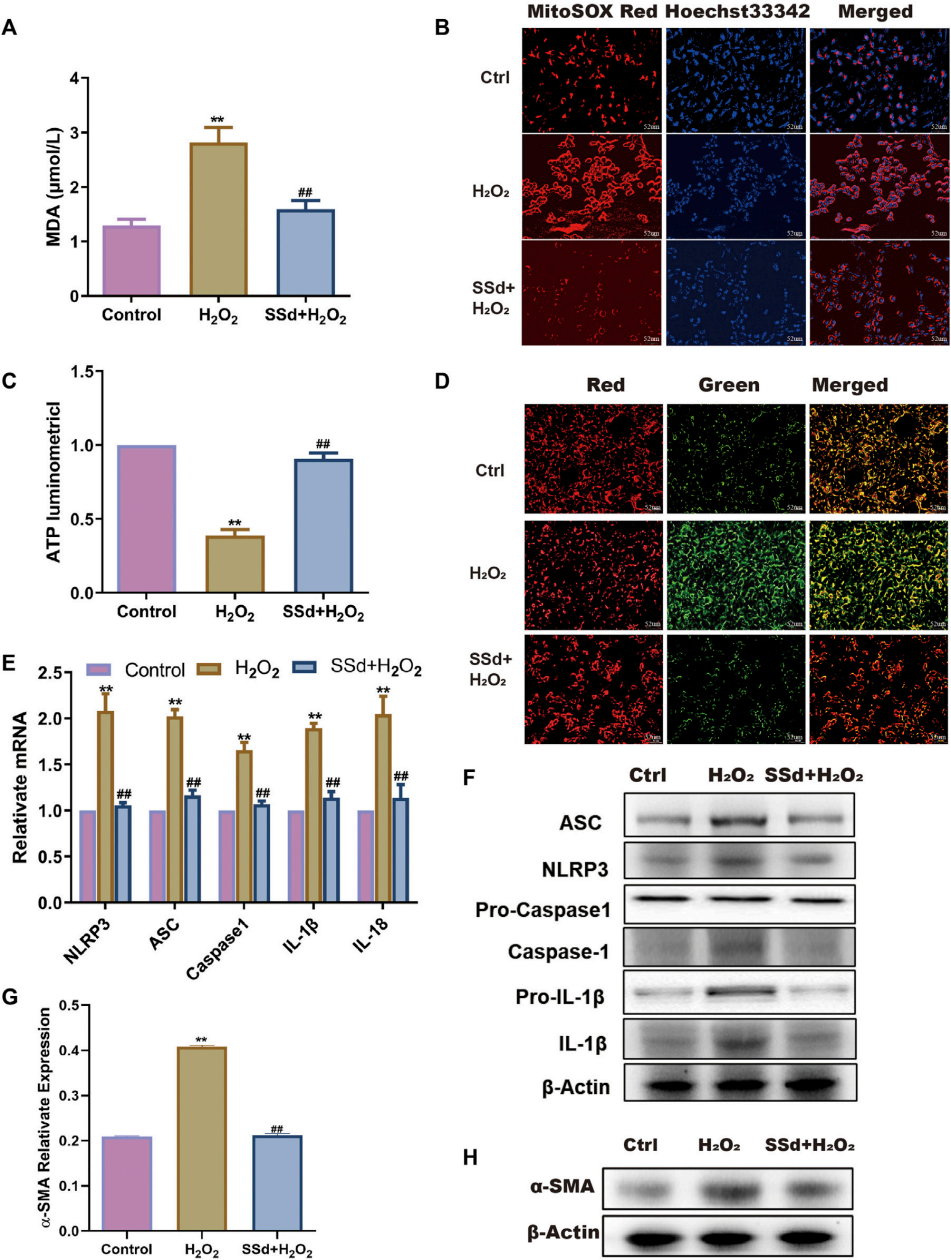
SSd alleviated H_2_O_2_-induced mitochondrial dysfunction and NLRP3 activation in HSCs **(A)** the content of MDA in HSCs after SSd intervention combination with H_2_O_2_ or not, *n* = 6. **(B)** MitoSOX Red fluorescence images (the heavier the red, the more damage, scale bar, 52 μm). **(C)** ATP content, shows the degree of damage to mitochondria in HSCs, *n* = 6. **(D)** JC-1 fluorescence images (the more JC-1 monomers with green, the more damage to mitochondria, scale bar, 52 μm). **(E,F)** RT-PCR and WB was employed to examine the expression of inflammasome, *n* = 3. **(G,H)** α-SMA protein expression, *n* = 3. Data are expressed in mean ± SEM; **p < 0.01, H_2_O_2_ treated group versus control group; ##*p* < 0.01, H_2_O_2_+SSd group versus H_2_O_2_ treated group.

To investigate whether SSd can protect the mitochondrial functions of activated HSCs *in vitro*, activated HSCs were treated with SSd. It was found that both MDA content and mitochondrial ROS level in HSCs were significantly decreased after SSd treatment (Figures 3A,B). Conversely, both intracellular ATP content and the mitochondrial proportion of JC-1 monomers with red fluorescence were increased after SSd treatment (Figures 3C,D). The effects of SSd treatment on the expression of NLRP3 inflammatory and fibro-genic proteins in activated HSCs were also examined. The results showed that SSd inhibited the expression of α-SMA, NLRP3 inflammasome, pro-IL-1β, IL-1β, and IL-18 (Figures 3E–H). Collectively, these results indicated that SSd alleviated H_2_O_2_-induced mitochondrial dysfunctions, as well as in reducing the expression of inflammatory factors in HSCs.

### Deficiency of ERβ, But Not ERα, Blocked the Inhibitory Effects of SSd Against CCL_4_-Induced Mitochondrial Dysfunctions and NLRP3 Inflammasome Activation

**FIGURE 4:**
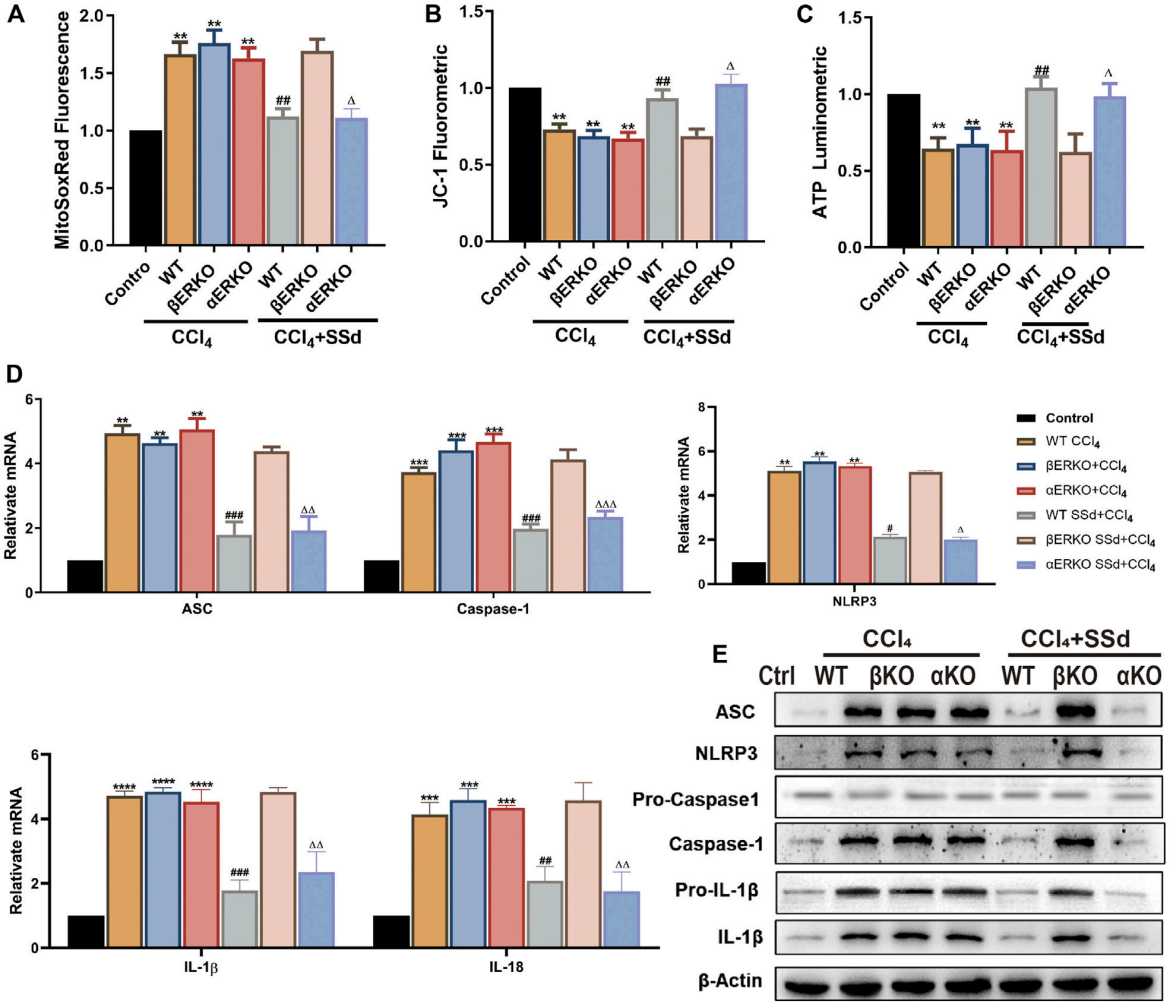
Deficiency of ERβ, but not ERα, blocked the inhibitory effect of SSd against CCl_4_-induced mitochondrial dysfunction and NLRP3 activation in the liver **(A)**. ROS fluorescence expression in the CCl_4_-induced ERKO mouse model combine with SSd or not, *n* = 6. **(B)** the mitochondrial JC-1 fluorescence expression in liver tissue, *n* = 6. **(C)** ATP content, *n* = 6. **(D,E)** inflammasome complex expression was tested by western blot experiment and PT-PCR in each group, *n* = 3. Data are expressed in mean ± SEM; **p < 0.01, ***p < 0.001, ****p < 0.0001, CCl_4_ treated versus control in WT and ERKO mice; ^##^ *p* < 0.01, ^###^ *p* < 0.001, ^####^ *p* < 0.0001, SSd + CCl_4_ group versus CCl_4_ group in WT mice; ^Δ^P <0.05, ^ΔΔ^P <0.01, ^ΔΔΔ^P <0.001, ^ΔΔΔΔ^P <0.0001, SSd + CCl_4_ group versus CCl_4_ group in αERKO mice.

Mitochondrial damages were also present in the CCL_4_-induced ERKO mice model. Compared with wild-type mice, mitochondrial ROS production was significantly upregulated, while the proportion of JC-1 monomers with red fluorescence and ATP content in the mitochondria were decreased in wild-type and ERKO mice treated by CCL_4_ (Figures 4A–C). After intervention with SSd, the above changes were reversed in wild-type and αERKO mice, but not in βERKO mice (Figures 4A–C). NLRP3 related mRNA and proteins were further examined in the liver of wild-type and ERKO mice treated with CCL_4_ alone or in combination with SSd. The results indicated that SSd effectively prevented CCl_4_-induced increase in NLRP3 inflammasome, pro-IL-1β, IL-1β, and IL-18 levels in wild-type mice and αERKO mice, but not in βERKO mice (Figures 4D,E).

### Deficiency of ERβ, But Not ERα, Blocked the Inhibitory Effect of SSd Against CCL_4_-Induced Liver Injury

**FIGURE 5:**
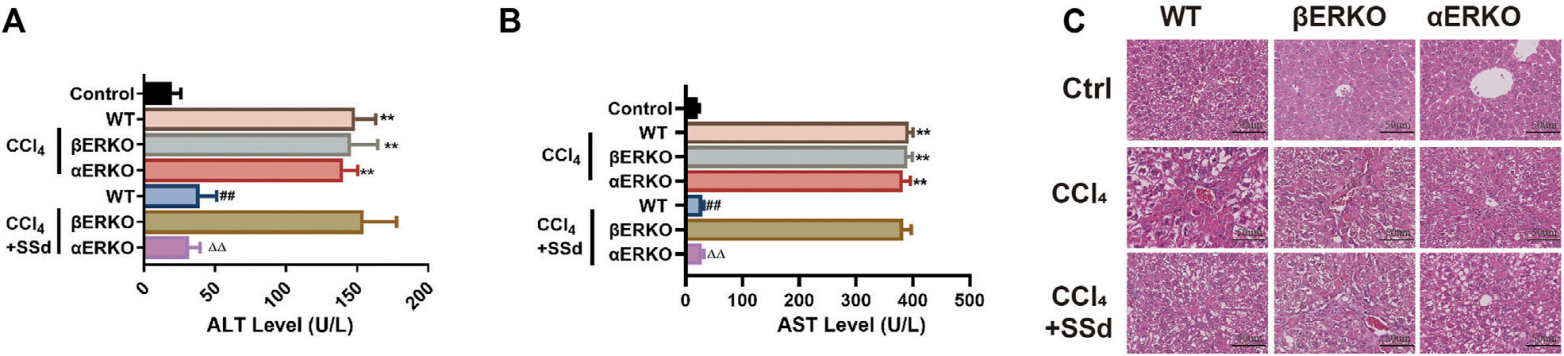
Deficiency of ERβ, but not ERα, blocked the inhibitory effect of SSd against CCL_4_-induced liver injury **(A,B)** plasma ALT and AST levels were tested after CCl_4_ treatment in WT and ERKO mice treated with SSd or without SSd, *n* = 6. **(C)** liver injury was detected by HE (×200; scale bar: 50 μm). Data are expressed in mean ± SEM; **p < 0.01, CCl_4_ treated versus control in WT and ERKO mice; ^##^ *p* < 0.01, SSd + CCl_4_ group versus CCl_4_ group in WT mice; ^ΔΔ^P <0.01, SSd + CCl_4_ group versus CCl_4_ group in αERKO mice.

SSd has estrogen-like effects *in vivo* and *in vitro*, and is an estrogen receptor modulator (Wang et al., 2010). It was hypothesized that SSd would have a therapeutic effect on liver injure and fibrosis. After CCl_4_ administration, increased ALT and AST activities were observed in both wild-type and ERKO mice, which could be significantly inhibited by SSd intervention in both wild-type and αERKO mice, but not in βERKO model mice (Figures 5A,B). Additionally, the H&E staining results revealed that SSd significantly reduced the necrotic area and the number of inflammatory cells in the liver of wild-type and αERKO liver fibrosis mice compared with the control group (Figure 5C). However, these changes were not observed in βERKO liver injure mice, suggesting that SSd blocked the inhibitory effects of SSd against CCL_4_-induced liver injury through the ERβ pathway.

### Deficiency of ERβ, But Not ERα, Blocked the Inhibitory Effect of SSd Against CCL_4_-Induced Liver Fibrosis

**FIGURE 6:**
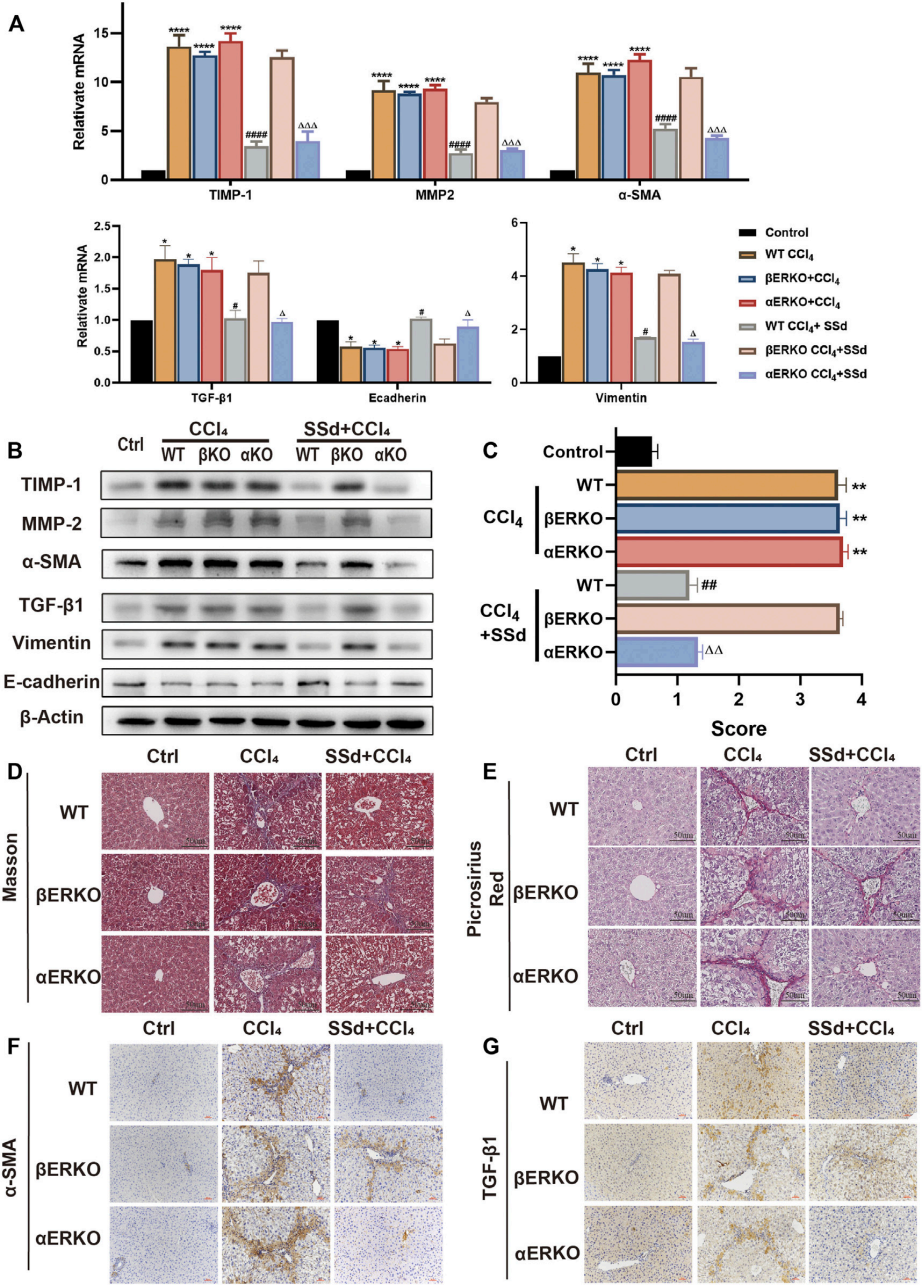
Deficiency of ERβ, but not ERα, blocks the inhibitory effect of SSd against CCl_4_-induced liver fibrosis **(A,B)**. α-SMA, TGF-β1, TIMP-1, MMP-2, E-cadherin, and Vimentin mRNA and protein expression were tested by RT-PCR and WB in liver tissues, *n* = 3. **(C)** liver fibrosis scores in each group, n = 6. **(D,E)** Liver fibrosis was detected by Masson staining, picric acid-Sirius staining (×200; scale bar: 50 μm). **(F,G)** IHC detection of the expression of α-SMA, TGF-β1 (×200; scale bar: 100 μm). Data are expressed in mean ± SEM; *p < 0.05, **p < 0.001, ****p < 0.0001, CCl_4_ treated versus control in WT and ERKO mice; ^#^ *p* < 0.05, ^##^ *p* < 0.01, ^####^ *p* < 0.0001, SSd + CCl_4_ group versus CCl_4_ group in WT mice; ^Δ^P <0.05, ^ΔΔ^P <0.01, ^ΔΔΔ^P <0.0001, SSd + CCl_4_ group versus CCl_4_ group in αERKO mice.

Consistent with the blood biochemistry results, SSd treatment failed to prevent liver fibrosis induced by CCl_4_ in βERKO mice. RT-PCR and WB analyses indicated that liver fibrosis-related factors (including α-SMA, TGF-β1, TIMP-1, MMP-2, and Vimentin) were significantly downregulated, while E-cadherin was significantly upregulated in wild-type and αERKO mice after SSd treatment (Figures 6A,B). Liver fibrosis scores of wild type mice and αERKO mice were also significantly lower than that of the control mice after SSd treatment (Figure 6C). Furthermore, liver sections from CCl_4_-induced wild-type and ERKO mice with or without SSd treatment were analyzed by Masson’s trichrome staining, Picric acid-Sirius red staining and IHC. It was observed that SSd effectively decreased collagen accumulation and the expression of α-SMA and TGF-β1 as compared with controls group in wild-type and αERKO mice (Figures 6D–G). No significant changes were observed in βERKO mice after SSd treatment, suggesting that SSd inhibited CCl_4_-induced liver fibrosis via the ERβ pathway.

### Specifically Antagonizing ERβ, But Not ERα, Blocked the Inhibitory Effects of SSd Against H_2_O_2_-Induced Mitochondrial Dysfunctions and NLRP3 Inflammasome Activation in HSCs

**FIGURE 7:**
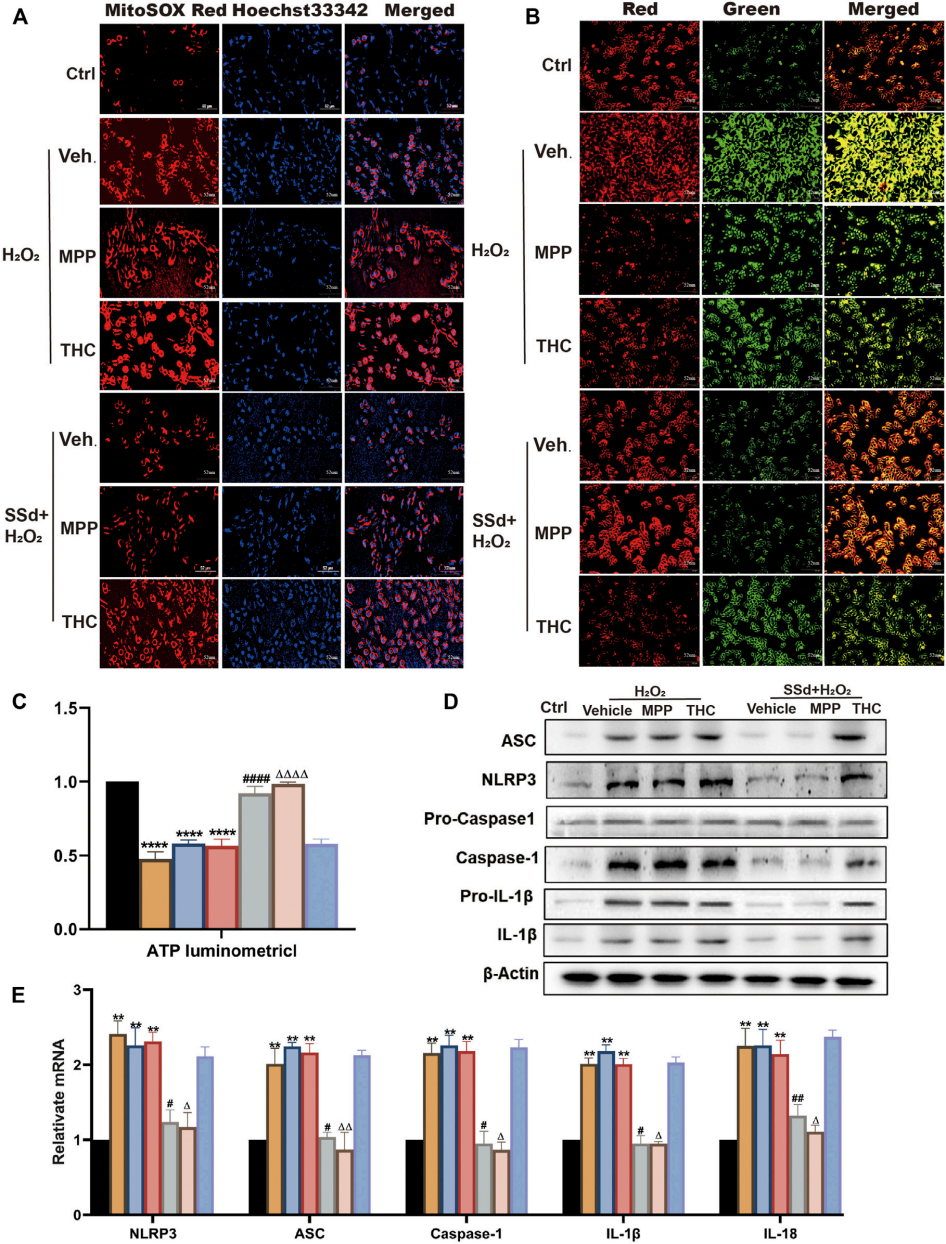
Specific antagonist of ERβ, but not ERα, blocked the inhibitory effect of SSd against H_2_O_2_-induced mitochondrial dysfunction and NLRP3 activation in HSCs **(A)**. ROS production in HSCs in each group in estrogen receptor blockers alone or in combination with SSd treated group in HSCs (normal or activated state) (Scale bar, 52 μm). **(B)** JC-1 membrane potential (Scale bar, 52 μm). **(C)** ATP content, *n* = 6. **(D,E)** inflammasome complex expression was tested by PT-PCR and western blot experiment. in each group, n = 3. Data are expressed in mean ± SEM; **p < 0.01, ****p < 0.0001, H_2_O_2_, H_2_O_2_ +MPP, and H_2_O_2_ +THC group versus control group; ^#^ *p* < 0.05, ^##^ *p* < 0.01, ^####^ *p* < 0.0001, SSd + H_2_O_2_ group versus H_2_O_2_ group; ^Δ^P <0.05, ^ΔΔ^P <0.01, ^ΔΔΔΔ^P <0.0001, MPP + SSd + H_2_O_2_ group versus MPP + H_2_O_2_ group.

To better understand the molecular mechanism underlying SSd’s effects on liver fibrosis, we attempted to link the ER pathway with the impaired activation of HSCs. First, ER blockers alone or in combination with SSd were used to treat HSCs (normal or activated). It was found that ER inhibitors alone had no significant effect on HSCs compared with the control group. Interestingly, when combined with SSd, ERα inhibitor MPP decreased ROS production, increased the proportion of JC-1 monomers with red fluorescence and intracellular ATP content, which were similar to the therapeutic effects of SSd alone (Figures 7A–C). However, treatment with the combination of SSd and ERβ inhibitor THC showed no significant effect (Figures 7A–C). Furthermore, the levels of NLRP3 related proteins were assessed, which shown that treatment with the combination of SSd and ERα blocker notably decreased the levels of NLRP3, pro-IL-1β and IL-1β proteins, the degrees of which were comparable to those caused by SSd treatment alone (Figure 7D). Consistent with these results, RT-PCR analysis revealed that the combination of ERα inhibitor and SSd inhibited the expression of inflammasome genes in HSCs (Figure 7E).

### Specifically Antagonizing of ERβ, But Not ERα, Blocked the Inhibitory Effects of SSd Against H_2_O_2_-Induced ECM Deposition and EMT in HSCs

**FIGURE 8:**
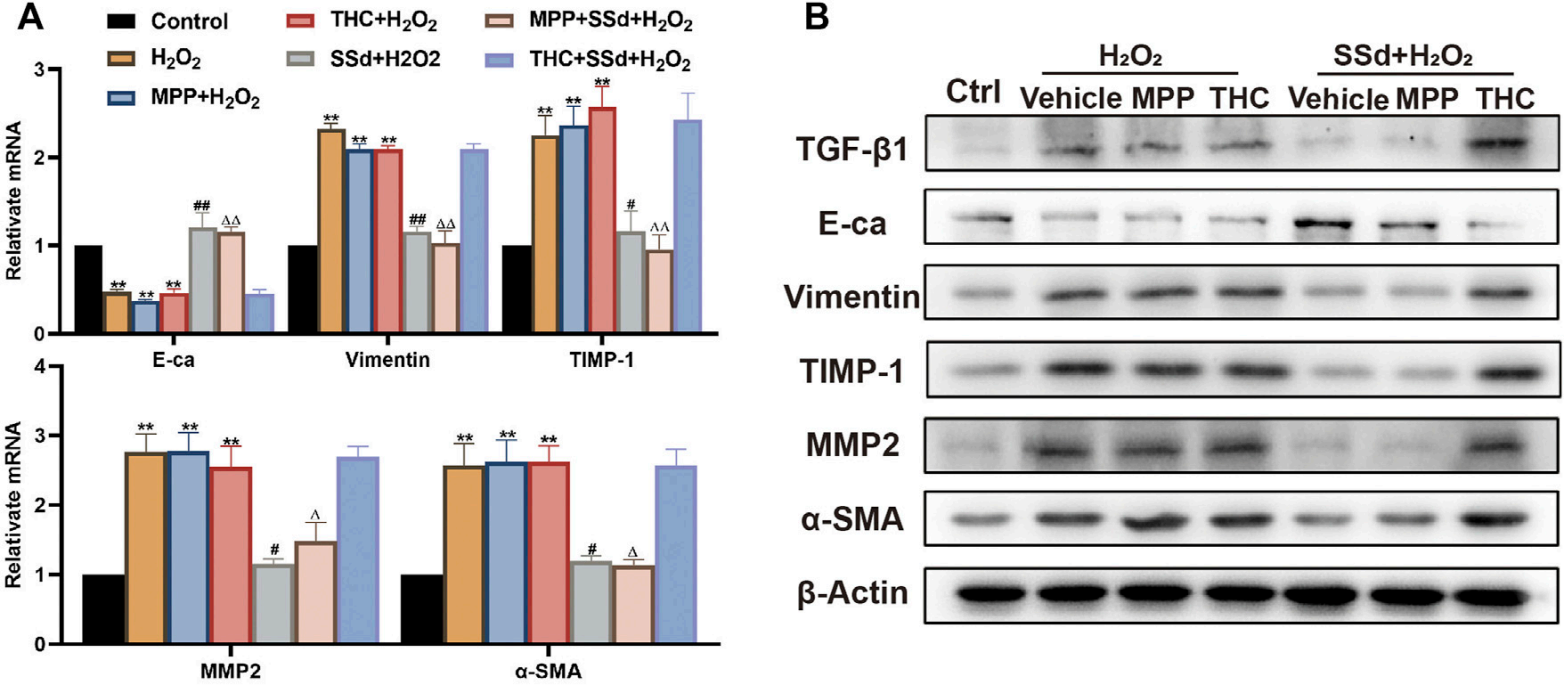
Specific antagonist of ERβ, but not ERα, blocked the inhibitory effect of SSd against H_2_O_2_-induced extracellular matrix deposition and EMT in HSCs **(A,B)** the indicators of extracellular matrix deposition and EMT (α-SMA, TGF-β1, TIMP-1, MMP-2, Vimentin, and Ecadherin) expression in HSCs were detected by RT-PCR and WB, *n* = 3. Data are expressed in mean ± SEM; **p < 0.01, H_2_O_2_, H_2_O_2_ +MPP, and H_2_O_2_ +THC group versus control group; ^#^ *p* < 0.05, ^##^ *p* < 0.01, SSd + H_2_O_2_ group versus H_2_O_2_ group; ^Δ^P <0.05, ^ΔΔ^P <0.01, MPP + SSd + H_2_O_2_ group versus MPP + H_2_O_2_ group.

To explore the biomechanism underlying SSd’s inhibitory effects on ECM deposition and EMT in activated HSCs, we tested the expression levels of relative indicators. The results showed that the combination of SSd and MPP significantly impaired the expression of α-SMA, TGF-β, TIMP-1, MMP-2, and vimentin, and improved the expression of E-cadherin in HSCs exposed to H_2_O_2_, while no therapeutic effect was observed in the SSd + THC intervention group (Figures 8A,B). Our results indicated that SSd-induced changes in expression of fibrosis-related factors depended on the activation of the ERβ pathway.

## Discussion

Our research confirmed the important role of SSd in the treatment of liver fibrosis. First, the application of SSd reduced mitochondria damage, activation of the NLRP3 inflammasome and inhibited HSCs activation and proliferation and liver injure and fibrosis. Second, the ERβ pathway played an important role in anti-liver fibrosis effects about SSd.

Subcutaneous CCl_4_ administration has been successfully applied to induce liver injury and cirrhosis, with histological changes and serum ALT levels being similar to those of human hepatic diseases. Persistent hepatocyte damages, excessive ECM deposition, and abnormal proliferation of connective tissue in the liver can lead to liver fibrosis (Hernandez-Gea and Friedman, 2011). HSCs activation, a key event in the occurrence of liver fibrosis, is the source of myofibroblasts and the final target of various fibrosis-inducing factors (Mederacke et al., 2013; Ray, 2014). Fortunately, EMT can be reversed into mesenchymal-epithelial transformation (MET), promoting the repair and reversal of hepatic fibrosis (Cicchini et al., 2015; Yang et al., 2020). Our experimental results showed that SSd intervention accelerated the degradation of deposited ECM, inhibited and reversed the process of EMT, and had anti-fibrosis effects. Early interventions can effectively alleviate liver fibrosis. Radix Bupleuri, the dry root of *Bupleurum Chinense* DC, has been characterized as a hepatoprotective herbal medicine that can treat various liver diseases, such as chronic hepatic inflammation, liver cirrhosis, hepatocellular carcinoma, and viral hepatitis (Li et al., 2018; Tian et al., 2019). SSd is one of the pharmacologically active compounds of Bupleurum. Recently, the anti-inflammatory, anti-fibrotic, anti-tumor, and hepatoprotective effects of SSd observed both *in vivo* and *in vitro* have attracted widespread attention (Xie et al., 2012; Yuan et al., 2017; Cui et al., 2019). A previous study showed that SSd could prevent hepatic inflammation and liver injury in acetaminophen-induced mice (Liu et al., 2014). The study showed that SSd reduced the infiltration and proliferation of CD8^+^ T cells, increased the expression of anti-inflammatory cytokines, inhibited the expression of TGF-β1, and ultimately alleviated inflammation and fibrosis in a mouse model of glomerulonephritis (Li et al., 2005). Furthermore, studies have shown that SSd can inhibit the activation of HSCs by regulating intracellular inflammatory responses, thereby eventually inhibiting liver fibrosis (Chen et al., 2013). Our previous studies also confirmed that SSd had a protective effect on CCl_4_-induced acute liver injury (Chen et al., 2020). CCl_4_-induced chronic liver injury and liver fibrosis model mice were treated with SSd, and found that liver injury and liver fibrosis were significantly alleviated by SSd treatment compared with the control group.

Activation of the NLRP3 inflammasome has been shown to cause liver injury and hepatic fibrosis in various acute and chronic liver diseases (Wree et al., 2014). Key components of a functional NLRP3 inflammasome are NLRP3, the adaptor protein ASC and caspase-1. NLRP3 recruits ASC and procaspase-1, which results in caspase-1 activation and processing of cytoplasmic targets, including the pro-inflammatory cytokines IL-1β and IL-18, whose purpose being to drive the inflammatory process and restore homeostasis. Mostly, priming is provided by signals of tissue damage activation downstream of pattern recognition receptors and is thought to be required both for NLRP3 and pro-IL-1β induction (Haneklaus and O'Neill, 2015). Active caspase-1 cleaves the cytokines pro-IL-1β and pro-IL-18 into their mature and biologically active forms (Martinon et al., 2002). Maria et al. introduced an NLRP3^−/−^ mouse model and confirmed that the NLRP3 inflammasome could directly activate HSCs and trigger liver fibrosis (Inzaugarat et al., 2019). Our experimental results confirmed that the NLRP3 inflammasome was overexpressed and activated during the process of liver fibrosis, suggesting that the increased levels of NLRP3 inflammasome proteins were associated with liver injury, liver fibrosis and HSCs activation. According to a previous study, mitochondrial ROS is the main activator of the NLRP3 inflammasome (Scambler et al., 2019). ROS can induce chronic inflammation and fibrous hyperplasia, leading to the activation of HSCs and finally promoting fibrosis (Dickie et al., 2012; Chen et al., 2019). In addition, oxidative stress significantly contributes to HSCs activation and fibrosis (Lin et al., 2020). Consistent with the findings in literature, our analyses of mitochondrial functions *in vivo* and *in vitro* confirmed that the production of mitochondrial ROS increased in CCl_4_-induced liver fibrosis mice and H_2_O_2_-induced activated HSCs. Therefore, reducing oxidative stress and eliminating ROS have been promising treatment strategies for liver fibrosis. SSd possesses anti-oxidative stress effects. According to a previous study, SSd could prevent oxidative damage, oxidative stress, and cytotoxicity in neural cells (Lin et al., 2016). SSd can reverse the impaired hepatic activity of superoxide dismutase, improve liver antioxidant capacity, inhibit lipid peroxidation, eliminate ROS, and ultimately prevent oxidative stress and liver injury (Fan et al., 2007). It was found that SSd significantly protected HL-7702 cells against CCl_4_-mediated oxidative stress and inhibited NLRP3 inflammasome-induced inflammation (Lin et al., 2018). Consistent with these findings, our study also confirmed that mitochondrial oxidative stress could activate HSCs and the NLRP3 inflammasome, which played an important role in the development of liver fibrosis. In this study, it was also focused on the correlation between SSd treatment and activation of the ROS/NLRP3 inflammasome axis. It was found that SSd treatment inhibited mitochondrial oxidative stress, reduced the production of mitochondrial ROS, downregulated the expression of NLRP3 inflammasome proteins and pro-IL-1β, IL-1β, and IL-18 induction, and eventually protected liver from injury and fibrosis.

Estradiol acts by binding to and activating different subtypes of estrogen receptors, including the two classical receptors ERα and ERβ, and the G protein-coupled estrogen receptor on the plasma membrane (Revankar et al., 2005; Dahlman-Wright et al., 2006). ERα and ERβ receptors can be detected in many tissues, but their distributions and expression levels are different. In liver cells, ERα is the predominant ER subtype, while ERβ is expressed in a variety of tissues during human fetal development; HSCs have been demonstrated to possess functional ERβ but not ERα; these findings suggest different organ-specific roles for the ERs (Brandenberger et al., 1997; Zhou et al., 2001; Gao et al., 2008). Some studies have shown that the function of E2 in preventing liver inflammation is mediated by ERα (Evans et al., 2002). Preclinical findings in rodents demonstrate that endogenous estradiol can prevent steatosis, insulin resistance, and fibrosis in liver diseases by activating the ER signal pathways (Grossmann et al., 2019). ERβ2 can lead to suppression of the ERα signaling (Zhao et al., 2007). ERβ may inhibit EMT (Mak et al., 2010) and effectively improve cirrhosis with portal hypertension in ovariectomized rats (Zhang et al., 2016). Zhang et al. found that selective agonists for ERβ exert antifibrogenic effects by inhibiting the activation and proliferation of HSCs (Zhang et al., 2018). These effects were obviously mediated by ERβ, but not ERα or GPER. SSd has estrogen-like effects *in vivo* and *in vitro*, and is an ER modulator (Wang et al., 2010). Liu et al. demonstrated that SSd improved memory in rats by modulating ERα activation, but not E2 level or ERβ activity, in the hippocampus (Liu et al., 2019). Que et al. found that SSd could inhibit oxidative stress-induced activation of HSCs, an effect that depended on the regulation of ERβ (21). Therefore, it can be seen that SSd activates estrogen ligands in difference organs and tissues with different functions. To this end, we introduced ERKO mice to verify that the anti-fibrosis effects of SSd were mediated by the ERβ pathway.

Studies have shown that E2 is a strong endogenous antioxidant that inhibits oxidative stress-induced lipid peroxidation and ROS production in rat liver mitochondria (Yagi, 1997; Omoya et al., 2001). Simvastatin could activate the expression of ERα and ERβ, thereby inhibiting the activation of the NLRP3 inflammasome and decreasing the levels of pro-inflammatory mediators (Menze et al., 2021). 17β-estradiol can reduce NLRP3 inflammasome activation to abrogate airway inflammation (Cheng et al., 2019). However, it has also been reported that E2 significantly inhibited the malignant behaviors of hepatocellular carcinoma cells through E2/ERβ pathway-mediated upregulation of the NLRP3 inflammasome (Wei et al., 2015). So far, a variety of estrogen receptor modulators have been found, such as soybean isoflavones, lignans, stilbenes, resveratrol, ginsenosides and SSd (Chan et al., 2002; Dixon, 2004). However, studies have shown that phytoestrogens have different affinities and transactivating activities for ERα and ERβ. For example, genistein and coumestrol preferentially interact with and activate ERβ rather than ERα to mediate estrogenic effects (Gutendorf and Westendorf, 2001). In addition, a previous study showed that SSd could upregulate the expression of ERα in the hippocampus of ovariectomized rats, but had no significant effect on the expression of ERβ (Liu et al., 2019). Moreover, they can inhibit the expression of NLRP3, HSCs activation, and liver fibrosis by acting on different estrogen ligands. Soybean isoflavones can alleviate DSS-induced colitis by inhibiting the ERα pathway and downregulating the subsequent activation of the NLRP3 inflammasome (Gao et al., 2020). Phytoestrogen calycosin inhibits the activation, proliferation, and migration of HSCs induced by TGF-β1 via downregulating ERβ (Deng et al., 2018). Comprehensive studies have shown that the mechanism of interaction between estrogen regulators and ligands is complex, and a specific regulator may even have opposite effects under different circumstances. Therefore, we used ER knockout mice to carry out experiments, and obtained strong evidence to support that SSd has ERβ pathway-dependent anti-liver fibrosis effects, such as inhibiting the expression of NLRP3 inflammasome proteins. By treating HSCs with ERα and ERβ blockers and establishing αERKO and βERKO knockout liver fibrosis mouse models, we found that the experimental results obtained from mouse and cellular model groups were not significantly different from those obtained from the THC intervention group and the βERKO mouse group, while there were significant differences between the MPP intervention group, the αERKO mouse group and the control group. These findings suggested that SSd inhibited HSCs activation through the ERβ pathway, and alleviated liver injury and liver fibrosis by negatively regulating the ROS/NLRP3 inflammasome. Thus, we confirmed a new molecular mechanism underlying SSd’s anti-fibrosis effects—by negatively regulating the ROS/NLRP3 inflammasome through activating the ERβ pathway.

## Conclusion

Collectively, the results of our study demonstrate SSd suppresses hepatic injure and fibrosis by exerting inhibiting mitochondrial injury effect that inhibit the activation of NLRP3 inflammasome, which in turn leads to inhibition of HSCs activation and proliferation. This suggests SSd may be an effective therapeutic agent for the treatment of hepatic fibrosis.

## Data Availability

The original contributions presented in the study are included in the article/Supplementary Material, further inquiries can be directed to the corresponding author.

## Abbreviations

CCl4, carbon tetrachloride; ER-β, estrogen receptor β; ERKO, estrogen receptors knockout; HSCs, hepatic stellate cells; mtROS, mitochondrial reactive oxygen species; MitoSOX Red, MitoSOX Red mitochondrial superoxide indication; NLRP3, NLR family, domain containing protein 3; SSd, saikosaponind d.
