# Supplementary material for: Saikosaponin d Alleviates Liver Fibrosis by Negatively Regulating the ROS/NLRP3 Inflammasome Through Activating the ERβ Pathway
Source: Front Pharmacol. 2022 May 25;13:894981. doi: 10.3389/fphar.2022.894981 (PMC9174603; doi:10.3389/fphar.2022.894981)

**Supplementary materials**

**Supplementary Figure 1**

**
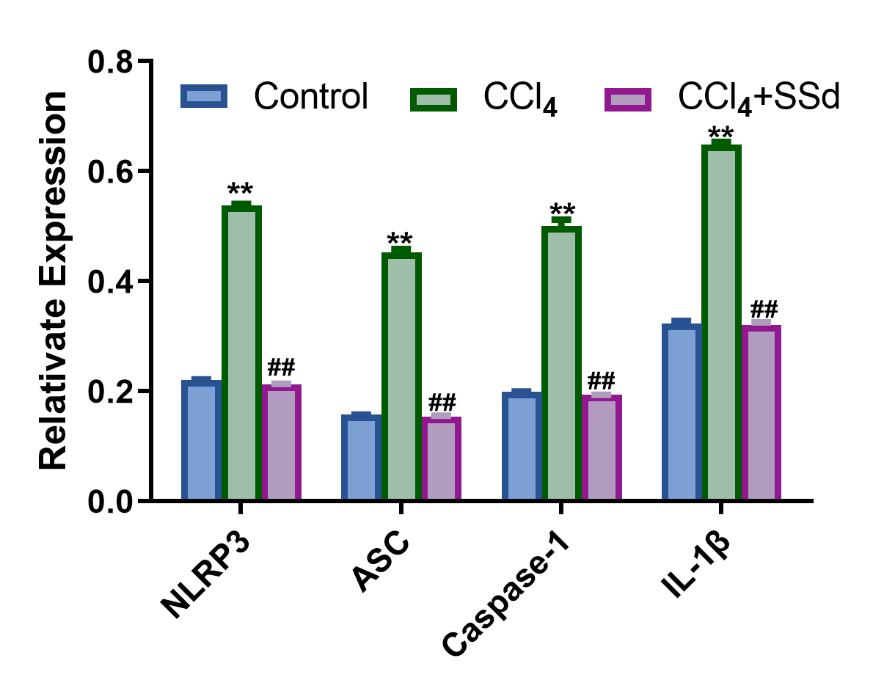
**

Supplementary Figure 1 NLRP3 inflammasome complex expression, n=3. Data are expressed in mean ± SEM; **P <0.01, versus control group; ^##^P <0.01, versus CCl_4_ treated group.

**Supplementary Figure 2**

**
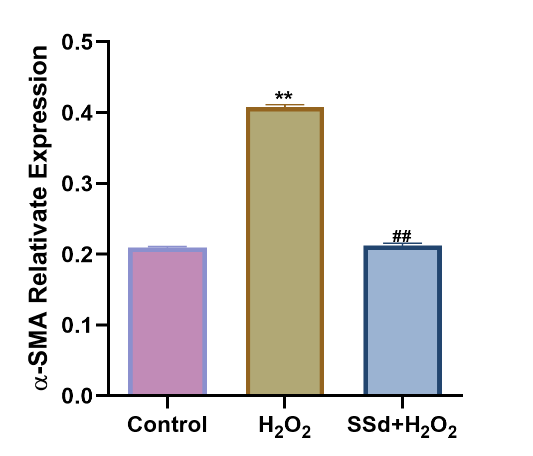

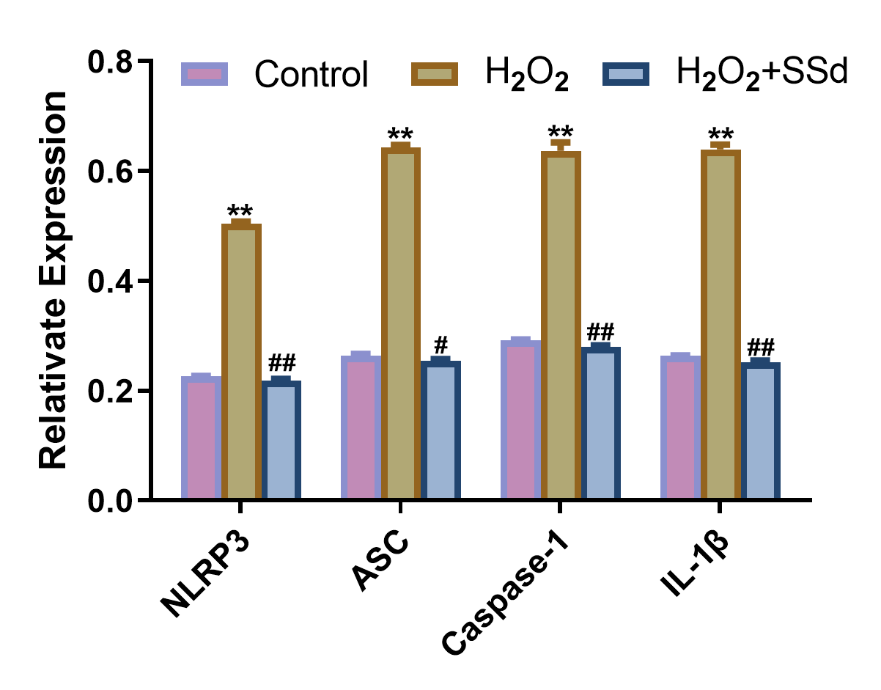
**

Supplementary Figure 2 NLRP3 inflammasome complex and α-SMA expression, n=3. Data are expressed in mean ± SEM; **P <0.01, versus control group; ^##^P <0.01, versus H_2_O_2_ treated group.

**Supplementary Figure 3.**


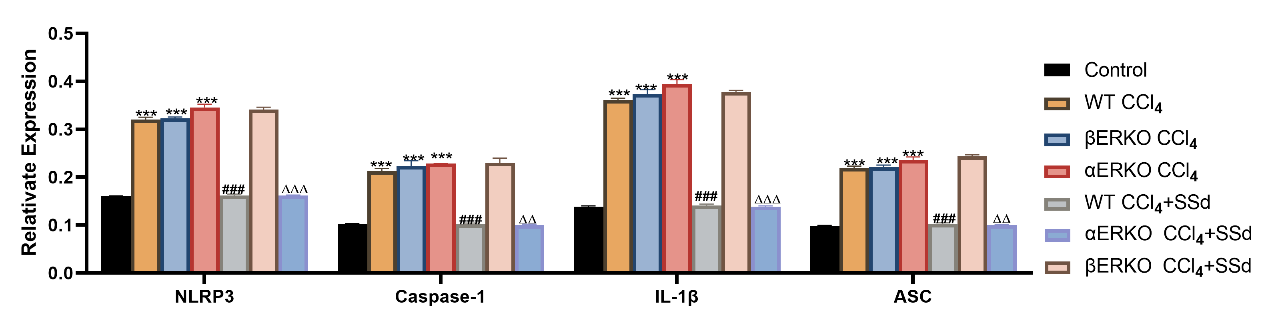


Supplementary Figure 3. NLRP3 inflammasome complex expression was tested by immunoprecipitation assay in the CCl_4_-induced WT and ERKO mice model combine with SSd or not. Data are expressed in mean ± SEM; *P <0.05 **P <0.01, ***P<0.001, ****P <0.0001, H_2_O_2_, H_2_O_2_ +MPP, H_2_O_2_ +THC group versus control group; ^###^P <0.001, SSd+ H_2_O_2_ group versus H_2_O_2_ group; ^Δ^P <0.05, ^ΔΔ^P <0.01, MPP+SSd+ H_2_O_2_ group versus MPP+H_2_O_2_ group

**Supplementary Figure 4.**


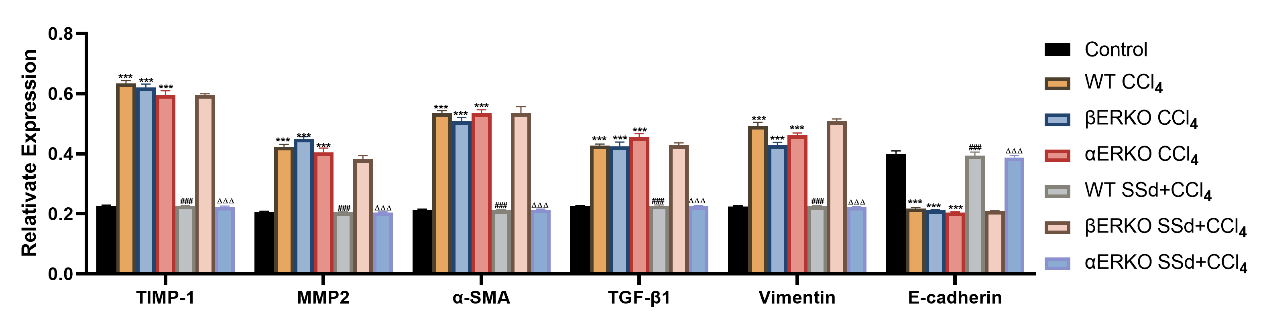


Supplementary Figure 4.α-SMA、TGF-β、TIMP-1、MMP-2、Ecadherin and Vimentin protein expression were tested after CCl_4_ treatment in WT and ERKO mice treated with SSd or without SSd. Data are expressed in mean ± SEM; *P <0.05, ***P <0.001,****P <0.0001, CCl_4_ treated versus control in WT and ERKO mice; ^##^P <0.01, ^####^P <0.0001, SSd+ CCl_4_ group versus CCL_4_ group in WT mice; ^ΔΔ^P <0.01, ^ΔΔΔ^P <0.0001, SSd+ CCl_4_ group versus CCl_4_ group in αERKO mice.

**Supplementary Figure 5**


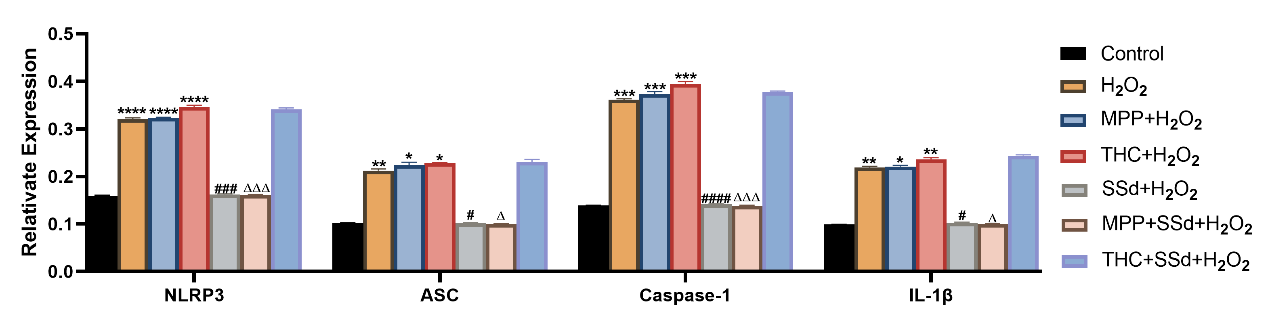


Supplementary Figure 5. NLRP3 inflammasome complex expression. Data are expressed in mean ± SEM; **P <0.01, ****P <0.0001, H_2_O_2_, H_2_O_2_ +MPP, H_2_O_2_ +THC group versus control group; ^#^P <0.05, ^##^P <0.01, ^####^P <0.0001, SSd+ H_2_O_2_ group versus H_2_O_2_ group; ^Δ^P <0.05, ^ΔΔ^P <0.01, ^ΔΔΔΔ^P <0.0001, MPP+SSd+ H_2_O_2_ group versus MPP+H_2_O_2_ group.

**Supplementary Figure 6**


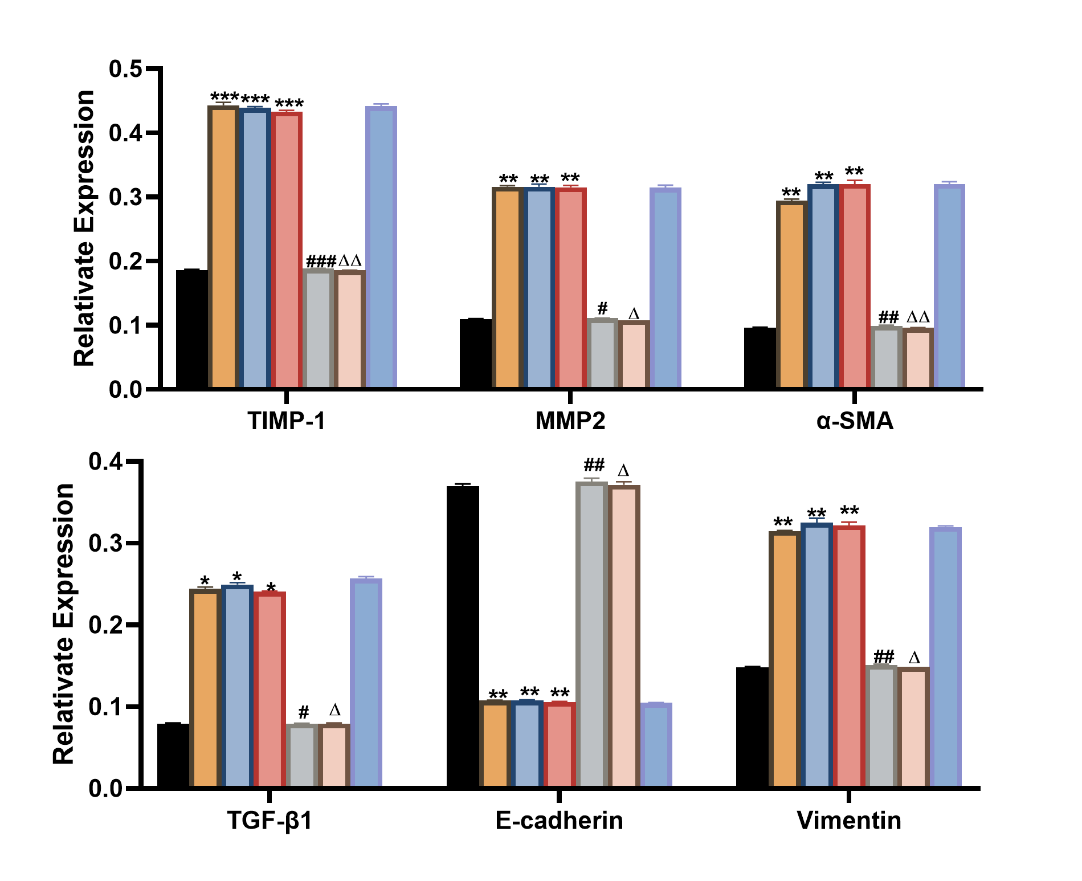


Data are expressed in mean ± SEM; **P <0.01, H_2_O_2_, H_2_O_2_ +MPP, H_2_O_2_ +THC group versus control group; ^#^P <0.05, ^##^P <0.01, SSd+ H_2_O_2_ group versus H_2_O_2_ group; ^Δ^P <0.05, ^ΔΔ^P <0.01, MPP+SSd+ H_2_O_2_ group versus MPP+H_2_O_2_ group.

**Table 1. Primary antibodies used in Western Blot and immunohistochemistry**


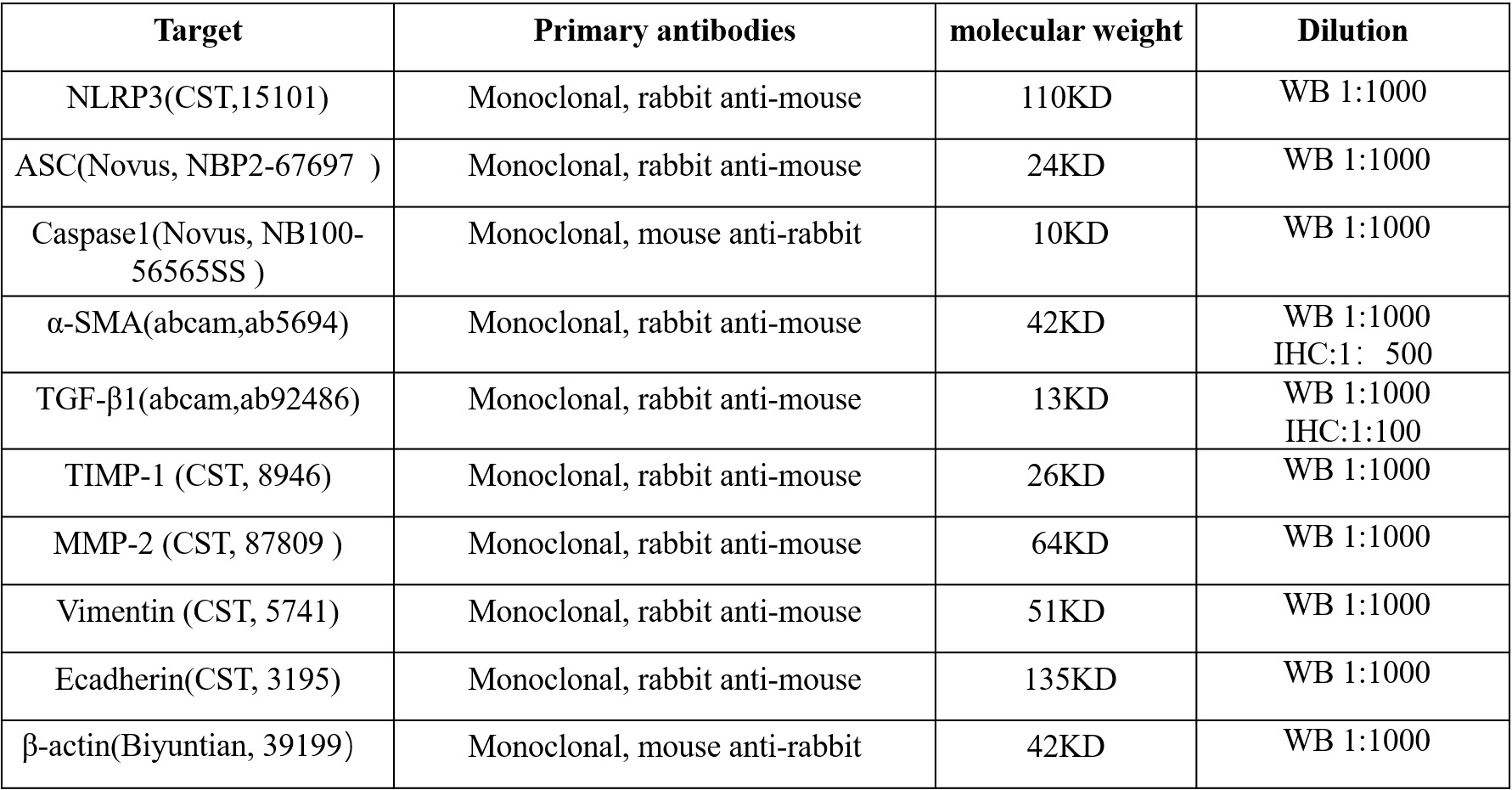


**Table 2. Sequences of primers (mouse) used in real time-PCR**


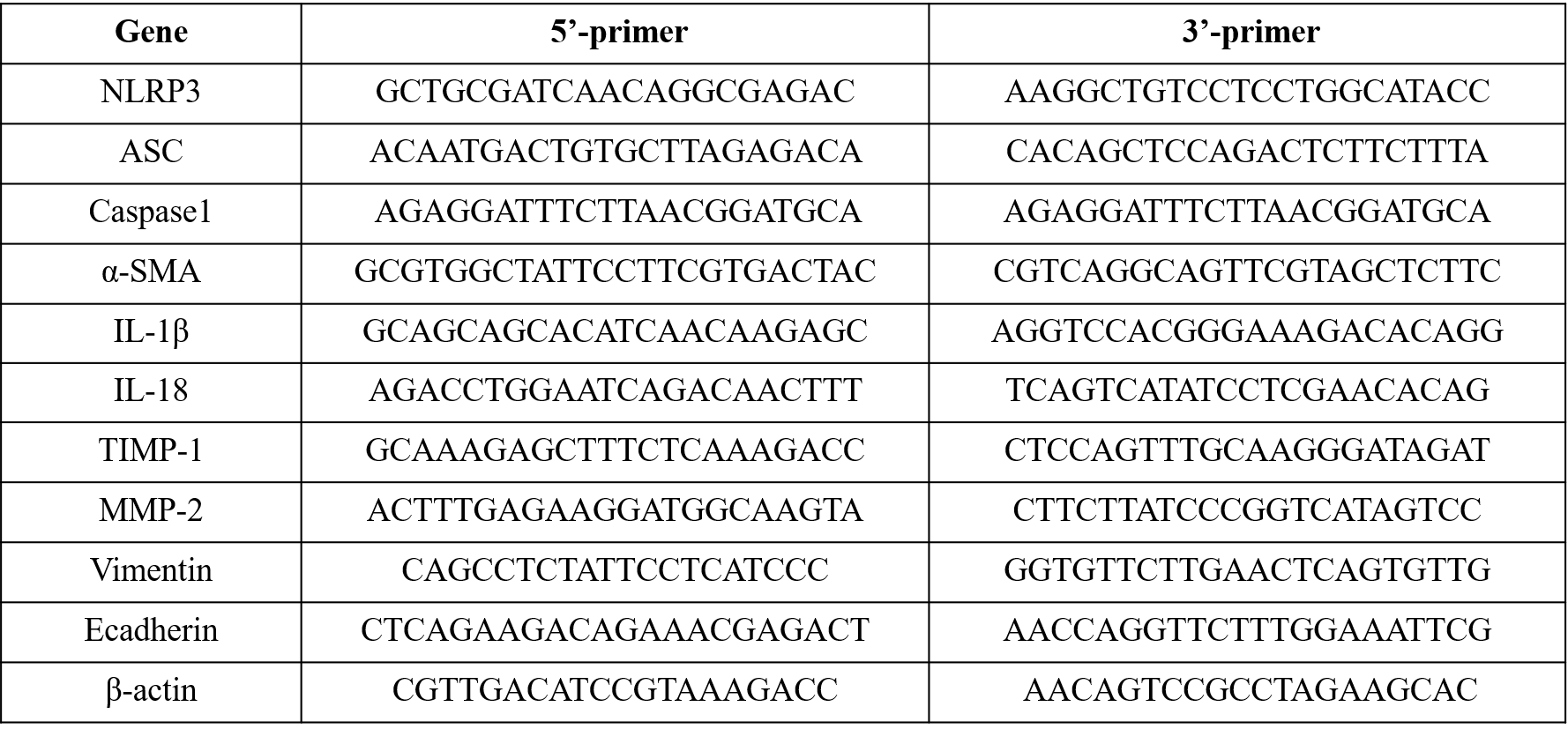


**Table 3. Sequences of primers (human) used in real time-PCR**


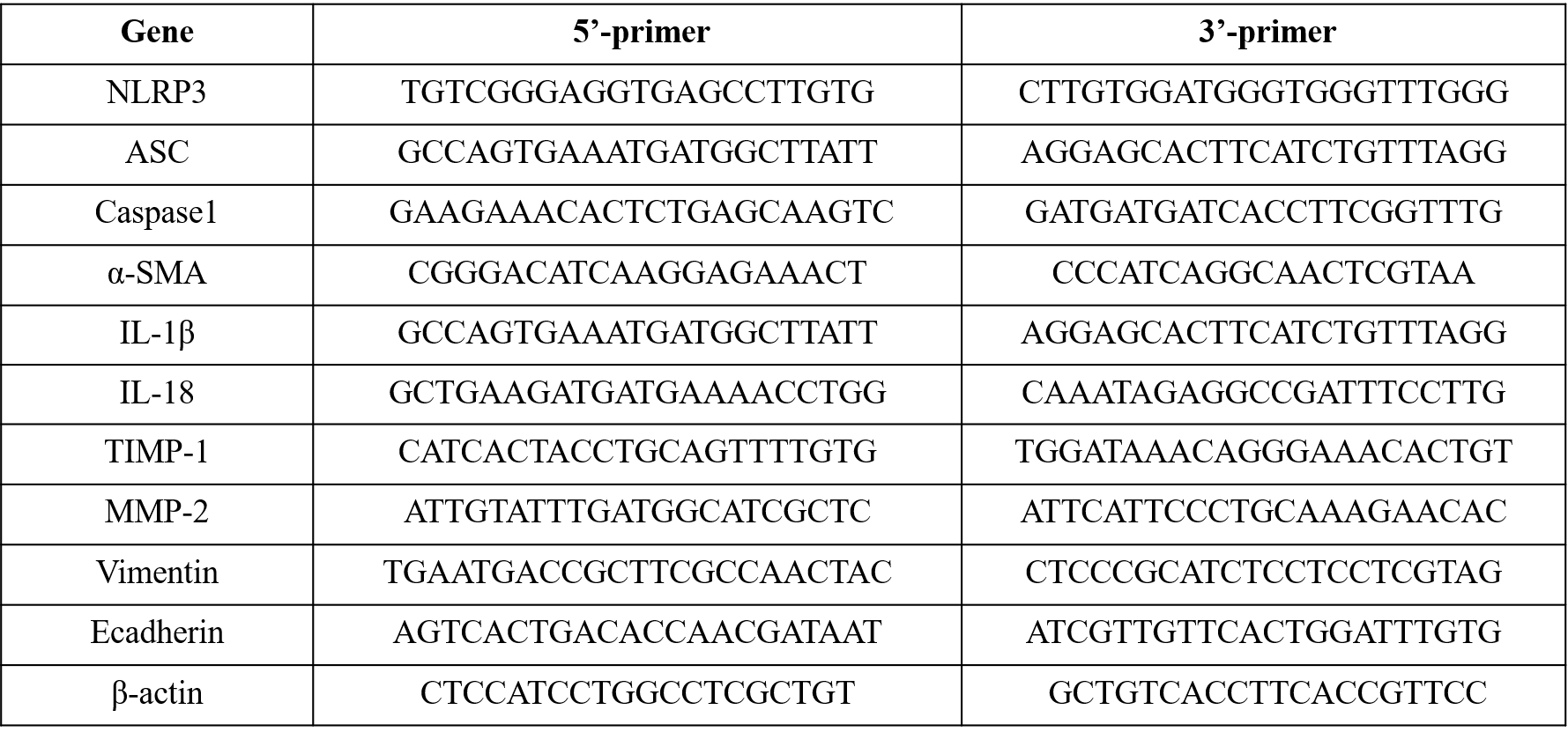

Supplement: Supplementary file 1 [file DataSheet1.docx]
